# Comparative effectiveness of palliative chemotherapy versus neoadjuvant chemotherapy followed by radical cystectomy versus cystectomy followed by adjuvant chemotherapy versus cystectomy for regional node‐positive bladder cancer: A retrospective analysis: KCSG GU 17‐03

**DOI:** 10.1002/cam4.2446

**Published:** 2019-07-29

**Authors:** Woo Kyun Bae, Hyo Jin Lee, Se Hoon Park, Jung Hoon Kim, Hee Jun Kim, Chi Hoon Maeng, Inkeun Park, Byeong Seok Sohn, Jung A. Kim, Kyung Hee Lee, Do Hyoung Lim, Hyun Chang, Sung Min Kim, Ho Young Kim, Hunho Song, Seungtaek Lim, Jae Ho Byun, Hyun Ae Jung

**Affiliations:** ^1^ Department of Hemato‐Oncology Chonnam National University Medical School Gwangju Republic of Korea; ^2^ Department of Internal Medicine Chungnam National University College of Medicine Daejeon Republic of Korea; ^3^ Division of Hematology‐Oncology, Department of Medicine, Samsung Medical Center Sungkyunkwan University School of Medicine Seoul Korea; ^4^ Department of Hemato‐Oncology Gyeongsang National University hospital Jinju Republic of Korea; ^5^ Department of Internal Medicine Chung‐Ang University College of Medicine Seoul Korea; ^6^ Division of Medical Oncology‐Hematology, Department of Medicine, College of Medicine Kyung Hee University Hospital Seoul Republic of Korea; ^7^ Division of Medical Oncology, Department of Internal medicine Gachon University Gil Medical Center Incheon Republic of Korea; ^8^ Department of Internal Medicine Inje University Sanggye Paik Hospital Seoul Republic of Korea; ^9^ Division of Medical Oncology‐Hematology, Department of Medicine, College of Medicine Kangdong Kyung Hee University Hospital Seoul Republic of Korea; ^10^ Department of Hemato‐Oncology, College of Medicine Yeungnam University Daegu Republic of Korea; ^11^ Department of Hemato‐Oncology Dankook University Hospital Cheonan Republic of Korea; ^12^ Department of Medical Oncology Catholic Kwandong University International St. Mary's Hospital Incheon Republic of Korea; ^13^ Division of Hematology‐Oncology, Department of Medicine, Samsung Changwon Sungkyunkwan University School of Medicine Changwon Korea; ^14^ Department of Internal Medicine, College of Medicine, Hallym University Medical Center Hallym University Anyang Korea; ^15^ Department of Internal Medicine, College of Medicine, Kangdong Sacred Heart Hospital Hallym University Seoul South Korea; ^16^ Department of Internal Medicine Wonju Severance Christian Hospital Wonju Korea; ^17^ Division of Oncology, Department of Internal Medicine, Incheon St. Mary's Hospital The Catholic University of Korea Incheon Korea

**Keywords:** bladder cancer, chemotherapy, lymph node, radical cystectomy

## Abstract

The regional lymph node‐positive bladder cancer was classified as stage IV in the AJCC 7th edition but was changed to stage IIIB in the 8th edition, revised in 2018. Among the various studies involving immune checkpoint inhibitors, groups that had only lymph node metastasis showed better outcomes than those with distant metastasis. Therefore, it is necessary to rethink the treatment strategy for lymph node‐positive bladder cancer. The aim of this study was to compare the treatment outcomes of chemotherapy, surgery, and combination therapy in patients with lymph node‐positive bladder cancer. From 1 January 2010 to 31 December 2015, patients with bladder cancer presenting local lymph node metastasis at the time of diagnosis were treated with a single treatment strategy, with either radical cystectomy or chemotherapy or with a combined strategy using both. Treatment outcomes were retrospectively analyzed on the basis of clinical indices and survival time. Out of 230 patients with bladder cancer, 44 (19.1%) were treated with palliative chemotherapy, 30 (13.0%) with neoadjuvant chemotherapy followed by cystectomy, 129 (56.1%) with cystectomy followed by adjuvant chemotherapy, and 27 (11.7%) with cystectomy alone. Median survival among all groups was 30.4 months. For each group, median overall survival was 19.3, 49.1, 42.6, and 11.2 months, respectively. This study represents an advancement in understanding the impact of clinical treatment patterns of lymph node‐positive bladder cancer through comparison of survival data of patients treated with different therapeutic strategies. Combined treatment resulted in better outcomes than did single treatments.

## Graphical abstract

This multi‐center retrospective study compared the outcome of different treatment modalities in node positive urothelial bladder cancer. The best survival rates were achieved in group treatment by neoadjuvant chemotherapy followed by radical cystectomy and radical cystectomy followed by adjuvant chemotherapy. This disease entity had not been studied homogenously until now. This study was the first to directly compare the results of various treatments in node‐ positive bladder cancer.

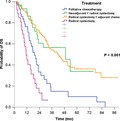

## 1 INTRODUCTION

The treatment for node‐positive bladder cancer, diagnosed using lymph node biopsy or image, is chemotherapy or chemotherapy combined with radiotherapy, in accordance with the NCCN and ESMO guidelines.^1^ Cisplatin combination chemotherapy such as gemcitabine and cisplatin (GP) or methotrexate, vinblastine, adriamycin, and cisplatin (MVAC) for node‐positive bladder cancer. MVAC has several side effects, including marrow suppression and mucositis, but is better tolerated in combination with G‐CSF. However, long‐term survival rates for locally advanced (ie, with extravesical and/or node‐positive disease) and metastatic disease remain dismal, with an overall survival (OS) of 9‐15 months and a 5‐year OS rate of 5% for the latter, even with the standard of care platinum‐based chemotherapy.^2^, ^3^, ^4^

In a population‐based study of patients with lymph node metastases, among 1739 patients with bladder cancer presenting lymph node involvement only 36.5% of patients received palliative chemotherapy.^5^ In a study from SEER data among 5201 patients who received cystectomy with lymphadenectomy for bladder cancer, 1260 (24.3%) patients had regional lymph nodes.^6^ Patients with lymph node metastases have an overall 5‐year survival rate of 31% after radical cystectomy. The number of lymph nodes removed at the time of cystectomy is associated with improved survival. In current clinical practice, although palliative chemotherapy is the standard of treatment, cystectomy with or without lymphadenectomy is frequently performed for lymph node‐positive bladder cancer.^5^, ^6^ The evidence of efficacy of neoadjuvant and adjuvant chemotherapy for treating bladder cancer after cystectomy is lacking because most neoadjuvant trials include patients with lymph node‐negative muscle‐invasive bladder cancer.^7^, ^8^, ^9^ Adjuvant cisplatin‐based chemotherapy is supported by a recent a large cohort analysis, but the results of this study are controversial.^10^

There were two randomized trials of first‐line immune checkpoint inhibitors in untreated metastatic urothelial cell cancer.^11^, ^12^ In a recent study using pembrolizumab as the first‐line treatment against cisplatin‐ineligible patients with locally advanced and unresectable or metastatic urothelial cell cancer, among 370 patients, 14% of them showed lymph node‐only metastasis and showed 47% of objective response rate.^12^ In a study of atezolizumab administered to 119 patients, 26% of them had lymph node‐only metastasis and showed a 32% objective response rate.^11^

In the AJCC 7th edition, lymph node metastasis is classified into stage IV, regardless of the degree or number of metastasis. Recently, the AJCC 8th edition revised in 2018, T1a‐T4a, N1, and M0 tumors were changed to stage IIIA and T1a‐T4a, N2‐N3, and M0 tumors were changed to stage IIIB. Previously, the group referred to as stage IV lymph node‐positive bladder cancer was divided into stage IIIA and stage IIIB.

As the biology of regional lymph node‐positive bladder cancer differs from that of metastatic bladder cancer and the classification of lymph node metastasis has recently changed, it is necessary to reconsider the best treatment strategy. Clinical data from various treatment strategies for treating regional lymph node‐positive bladder cancer are available. We have very few clinical available data comparing treatment outcomes for regional lymph node‐positive bladder cancer. Therefore, comparing the effectiveness of different treatment strategies, using chemotherapy, surgery, or a combination of the two is of critical importance. We performed a comparative analysis of four treatment strategies in regional lymph node‐positive bladder cancer: palliative chemotherapy, neoadjuvant chemotherapy followed by cystectomy, cystectomy followed by adjuvant chemotherapy, and cystectomy alone.

## 2 MATERIALS AND METHODS

### 2.1 Study subjects and data collection

This study is a multicenter, retrospective study that included patients with histologically confirmed urothelial cell carcinoma in bladder ranging from 2010 to 2015. This study was performed at 16 centers in South Korea. According to AJCC 7th stage classification criteria, they were all T, N1‐3, M0 tumors. The definition of regional positive lymph node is >1.5 cm as seen in an abdomen‐pelvis CT or MRI and located in the true pelvis or in the common iliac. Patients were older than 20 years and received one of the following four treatments: (a) palliative chemotherapy; (b) preoperative chemotherapy followed by radical cystectomy; (c) radical cystectomy followed by adjuvant chemotherapy; or (d) radical cystectomy.

### 2.2 Statistical analysis

All data cutoff for the analysis was 30 June 2018. To compare treatment patterns, patients were divided according to the clinical nodal stage and the chi‐squared test was used. The OS curve was calculated using the Kaplan‐Meier estimator and compared using the log rank test. OS is presented in all figures and tables as median values and two‐sided 95% confidence intervals (CIs). OS was defined as the first date of diagnosis until death resulting from any cause. Data were analyzed using SPSS‐19 (IBM SPSS Statistics. Inc).

### 2.3 Ethics statement

The protocol was approved by the Protocol Review Committee of the Korean Cancer Study Group (KCSG GU 17‐03) and by the Institutional Review Board at each participating institute. The trial was conducted in accordance with the Declaration of Helsinki.

## 3 RESULTS

### 3.1 Patient characteristics

During the study period, 230 patients diagnosed with regional lymph node‐positive bladder cancer from 18 member institutions of the Korean Cancer Study Group were included. During the study period, 44 patients received palliative chemotherapy, 30 received neoadjuvant chemotherapy followed by radical cystectomy, 129 received radical cystectomy followed by adjuvant chemotherapy, and 27 received radical cystectomy only (Table 1). According to the AJCC 7th edition stage classification criteria, 100 patients were N1 stage and 130 patients were N2‐N3 stage. For the neoadjuvant chemotherapy, 28 patients received the GP regimen, one patient received the MVAC regimen, and another received the cisplatin, methotrexate, and vinblastine (CMV) regimen. The median cycle of neoadjuvant chemotherapy before radical cystectomy was 2 (range: 1‐4). For adjuvant chemotherapy, all patients received GP, and the median cycle of chemotherapy was 3 (range: 1‐7). For first‐line palliative chemotherapy, 42 patients received GP, one patient received CMV regimen, and another received MVAC regimen.

**Table 1:** Baseline characteristics

Parameter	Number of patients	%
Age		
Median: 66.8 years old (range: 26.4‐86.1)		
Sex		
M	203	88.3%
F	27	11.7%
ECOG		
0‐1	195	84.8%
2	16	7.0%
Not available	19	8.2%
Clinical T stage		
T1	16	7.0%
T2	77	33.5%
T3	93	40.4%
T4	38	16.5%
Not available	6	2.6%
Clinical nodal stage		
N1	100	43.5%
N2‐N3	130	56.5%
Treatment		
Palliative chemotherapy	44	19.1%
Neoadjuvant (palliative chemo) + op	30	13.0%
Op + adjuvant (palliative chemo)	129	56.1%
Cystectomy +/− lymphadenectomy	27	11.7%

### 3.2 Treatment patterns by nodal stage

We evaluated treatment patterns dividing the patients by nodal stage (Table 2). In patients who had N1 stage, 8.0%, 19.0%, 62.0%, and 11.0% received palliative chemotherapy, neoadjuvant chemotherapy followed by radical cystectomy, radical cystectomy followed by adjuvant chemotherapy, and radical cystectomy only, respectively, while in the N2‐N3 group, the percentages were 27.6%, 8.7%, 51.2%, and 12.6%, respectively.

**Table 2:** Treatment patterns and outcomes by nodal stage

		Clinical N1 stage			Clinical N2‐N3 stage		
		Number of patient (%)	Median OS	*P*	Number of patients (%)	Median OS (mo)	*P*
Palliative chemotherapy	N = 43	8 (8.0%)	21.0	<.001	35 (27.6%)	19.3	<.001
Neoadjuvant followed by radical cystectomy	N = 30	19 (19.0%)	49.1		11 (8.7%)	27.7	
Radical cystectomy followed by adjuvant chemotherapy	N = 127	62 (62.0%)	45.9		65 (51.2%)	33.0	
Radical cystectomy	N = 27	11 (11.0%)	16.0		16 (12.6%)	11.0	

### 3.3 Overall survival by treatment

**Figure 1:**
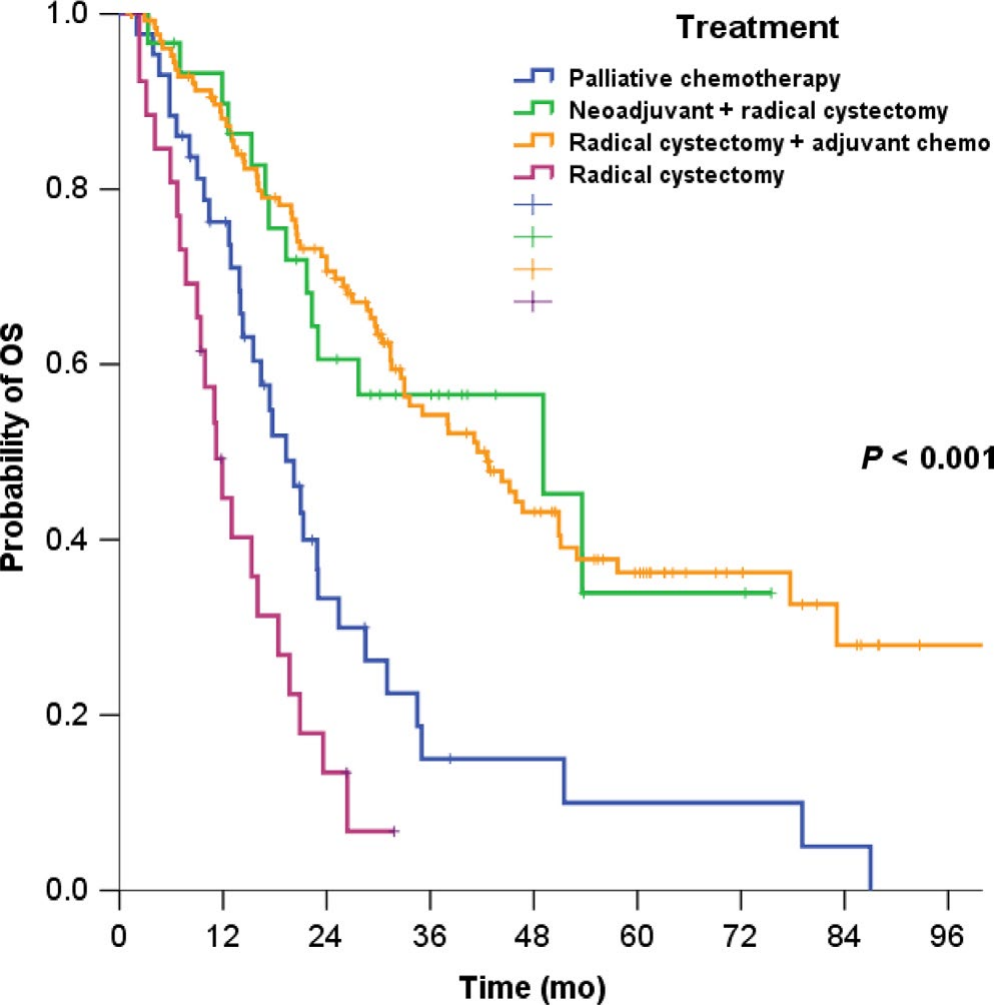
Survival curve by treatment in total population

The median OS for regional lymph node‐positive bladder cancer was 30.4 months (95% CI [lower‐upper], 25.3‐35.6). The median OS were 19.3 months (95% CI, 17.3‐24.3) for palliative chemotherapy, 49.1 months (95% CI, 20.7‐77.5) for neoadjuvant chemotherapy followed by radical cystectomy, 42.6 months (95% CI, 32.0‐53.2) for radical cystectomy followed by adjuvant chemotherapy, and 11.2 months (95% CI, 25.2‐35.6) for radical cystectomy only (*P* < .001) (Figure 1).

### 3.4 Overall survival by N stage

**Figure 2:**
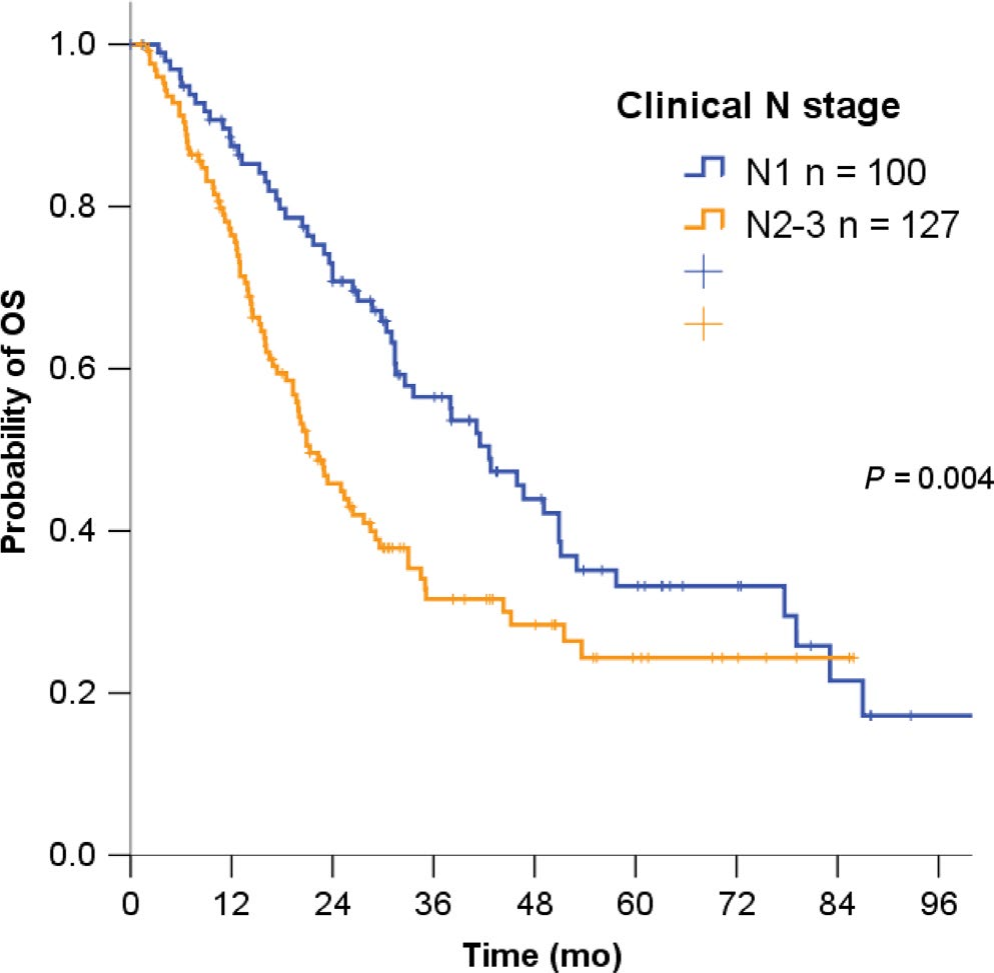
Survival curve by nodal stage

**Figure 3:**
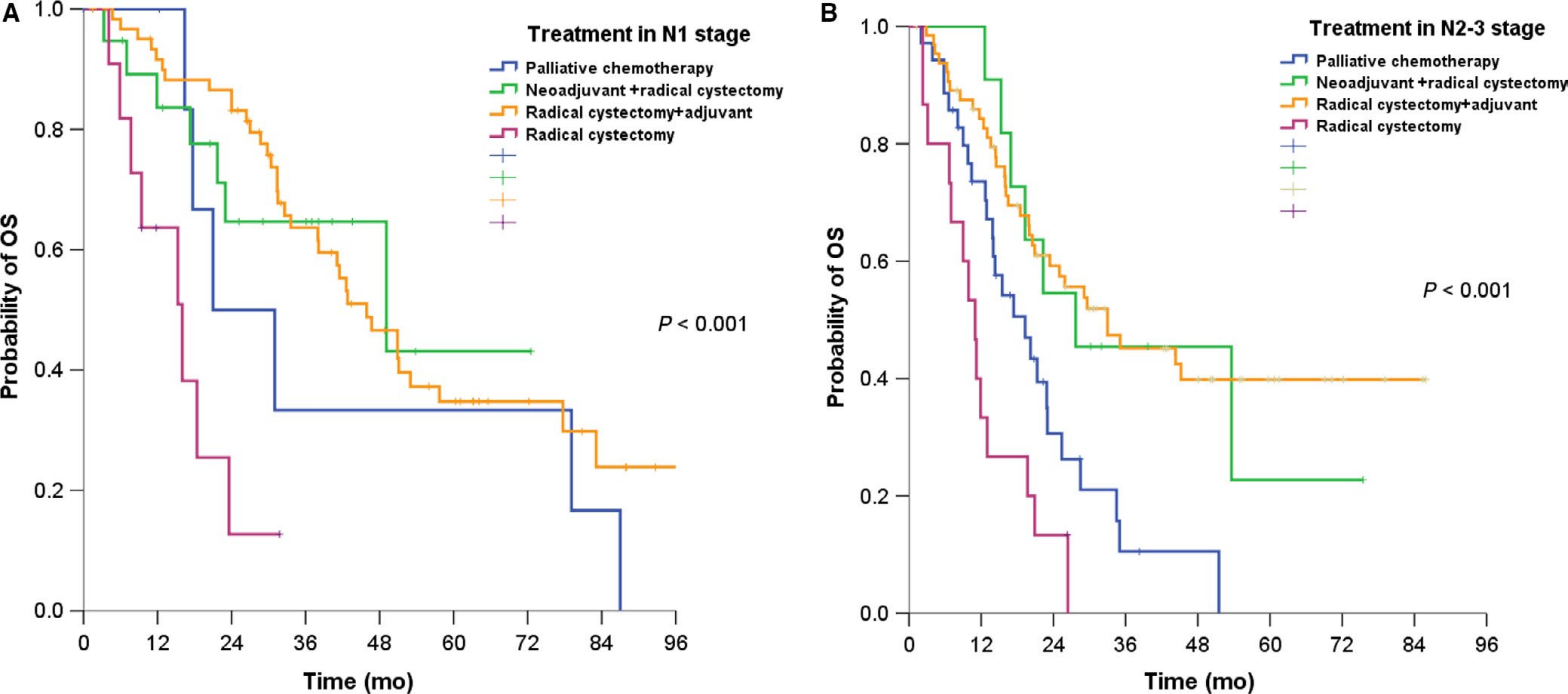
(A) Survival curve by treatment in N1 stage group. (B) Survival curve by treatment in N2‐N3 stage group

The median OS was 42.6 months (95% CI, 33.9‐51.3) for the N1 stage versus 21.3 months (95% CI, 16.9‐25.7) for N2‐N3 stage in bladder cancer (*P* = .004) (Figure 2). In the group with N1 stage, the median OS was 21.0 months for palliative chemotherapy, 49.1 months for neoadjuvant chemotherapy followed by radical cystectomy, 45.9 months for radical cystectomy followed by adjuvant chemotherapy, and 16.0 months for radical cystectomy only (*P* < .001) (Figure 3A). In the group with N2‐N3 stage, the median OS was 19.0 months for palliative chemotherapy, 27.7 months for neoadjuvant chemotherapy followed by radical cystectomy, 33.0 radical cystectomy followed by adjuvant chemotherapy, and 11.0 months for radical cystectomy only (*P* < .001) (Figure 3B).

## 4 DISCUSSION

The aim of this study was to compare different treatment strategies for regional lymph node‐positive bladder cancer with the aim of understanding the current clinical treatment patterns. While radical cystectomy for lymph node‐positive bladder cancer is possible, our results showed that neoadjuvant chemotherapy followed by radical cystectomy or radical cystectomy followed by postoperative chemotherapy is superior in terms of OS.

In 2016, there were 4361 new cases of bladder cancer in Korea.^13^ In a previous clinical trial of stage IV bladder cancer, the proportion of patients with any T, N1‐N3, M0 tumor grade was about 15%.^12^ Several guidelines indicated that palliative chemotherapy is the standard of treatment for regional lymph node‐positive bladder cancer. If tumor shrinkage is noted following the chemotherapy, radical cystectomy could be considered. Until now, no study has focused on the treatment outcome in regional lymph node‐positive bladder cancer. This type of cancer has been treated in the clinic on the basis of a previous study of muscle‐invasive bladder cancer. Recently, radical cystectomy with extended lymphadenectomy has been considered for curative intent for regional lymph node‐positive bladder cancer.^6^ However, the surgery‐only strategy showed poor outcomes and there was no study outcomes on neoadjuvant chemotherapy followed by radical cystectomy. Only muscle‐invasive and lymph node‐negative bladder cancer shows sensitivity to neoadjuvant chemotherapy.^7^, ^9^ Among our groups, 30 patients who received neoadjuvant chemotherapy followed by radical cystectomy showed the most favorable OS.

In bladder cancer, the knowledge of tumor biology and somatic alterations could lead to the discovery of novel therapeutic targets, such as the recently developed immune checkpoint inhibitors.^14^, ^15^ According to AJCC 7th edition classification criteria, bladder cancer with regional lymph node involvement was considered at stage IV, and thus surgery was not indicated. However, recent retrospective data showed favorable outcome for regional lymph node‐positive bladder cancer treated with surgery, and several surgeons perform radical cystectomy rather than administer the suggested palliative chemotherapy. New surgical techniques, such as extended lymphadenectomy including cephalad boundary at the aortic bifurcation or take off from the inferior mesenteric artery, which showed better outcomes were recently developed.^6^ In a study of immune checkpoint inhibitors, patients who had only regional lymph node metastasis showed better outcomes than those with distant metastasis, but these groups presented tumors with different biological origins (urothelial cancer).^11^, ^12^

In a previous observation cohort study,^5^ subsets of patients with regional lymph node‐positive bladder cancer showed durable disease control and long‐term survival. Moreover, compared to single modality including palliative chemotherapy or surgery alone, combined modality treatments showed better outcomes.

However, it is important to point out that this study has some limitations. The retrospective nature and observational study design lead to drawbacks including potential selection bias and unmeasured confounders, such as performance status indicator or other organ function parameters. In practice, the patients selected for combined radical cystectomy and chemotherapy would be generally fitter than the patients selected for palliative chemo or cystectomy only.

Patients who received concurrent chemoradiotherapy (CCRT) for bladder cancer were excluded. In bladder cancer, CCRT could be considered as bladder preservation treatment in node‐negative case if patients refused radical cystectomy or were not eligible for radical cystectomy. In a multicenter, randomized, phase three trial, combined CCRT showed better locoregional control than did radiotherapy alone for node‐negative, muscle‐invasive bladder cancer.^16^ Chemoradiotherapy after transurethral resection of muscle‐invasive, node‐negative bladder cancer showed 72% complete response, 64% of 10‐year local control, and 42% of 10‐year disease‐specific survival.^17^

In clinical practice, as physicians encounter patients with bladder cancer of varying stages, a multidisciplinary approach for the initial diagnosis could be advised and a combined therapy strategy for the treatment of regional lymph node‐positive bladder cancer should be considered.

This study showed that a combined therapy strategy to treat regional lymph node‐positive bladder cancer had better outcomes than those of single therapy strategy. For example, radical cystectomy followed by adjuvant chemotherapy compared with radical cystectomy alone showed better outcomes. To determine the optimal sequence for the combined therapy that includes chemotherapy and radical cystectomy, this study included 30 patients who received neoadjuvant chemotherapy followed by radical cystectomy and 127 patients who received radical cystectomy followed by adjuvant chemotherapy. If the extent of regional lymph node‐positive bladder cancer was ineligible for radical cystectomy initially, after at least two cycles of chemotherapy, reevaluation for radical cystectomy could be considered, according to the combined therapy approach. However, due to the small number of patients included in neoadjuvant chemotherapy, the comparison to the adjuvant group directly was limited.

## 5 CONCLUSIONS

Combined modality with radical cystectomy with perioperative chemotherapy showed better outcomes than single therapy strategies for regional lymph node‐positive bladder cancer. In this study, we also tried to find subsets of patients who were feasible to be treated with a combined therapy approach, using a multidisciplinary approach at initial diagnosis and during treatment.

## CONFLICT OF INTEREST

None declared.
